# Energy-resolved neutron imaging study of a Japanese sword signed by Bishu Osafune Norimitsu

**DOI:** 10.1038/s41598-024-79436-6

**Published:** 2024-11-14

**Authors:** Kenichi Oikawa, Yoshihiro Matsumoto, Kenichi Watanabe, Hirotaka Sato, Joseph Don Parker, Takenao Shinohara, Yoshiaki Kiyanagi

**Affiliations:** 1 J-PARC Center, Japan Atomic Energy Agency, 2-4 Shirakata, Tokai, Ibaraki 319-1195 Japan; 2 Comprehensive Research Organization for Science and Society, Neutron Science and Technology Center, 162-1 Shirakata, Tokai, Ibaraki Japan; 3 Faculty of Engineering, Kyushu University, 744 Motooka, Nishi-Ku, Fukuoka, 819-0395 Japan; 4 Faculty of Engineering, Hokkaido University, Kita-13 Nishi-8, Kita-Ku, Sapporo, Hokkaido 060-8628 Japan; 5 Japan Neutron Optics Inc, 20-5 Takeshima-Cho, Gamagori, Aichi 443-0031 Japan; 6 Prof. Emeritus, Hokkaido University, Kita-13 Nishi-8, Kita-Ku, Sapporo, Hokkaido 060-8628 Japan

**Keywords:** Bragg-edge transmission imaging, Energy-selective neutron tomography, Forge welding, Hardened structure, Japanese sword, Imaging techniques, Characterization and analytical techniques

## Abstract

Our research focuses on elucidating the crystallographic structure of Japanese swords in a nondestructive manner using the neutron imaging instrument RADEN at the Materials and Life Science Experimental Facility of the Japan Proton Accelerator Research Complex (J-PARC). We developed an analysis method combining wavelength-resolved Bragg-edge imaging and wavelength-selective neutron tomography with a new strategy and applied it to an approximately 45-cm blade length Japanese sword signed by Bishu Osafune Norimitsu. Computed tomography was performed, and the three-dimensional analysis captured the characteristic internal structure of *Kobuse*. Kobuse is the most famous steel-combining structure of Japanese swords, where an outer steel with high-carbon content (*Kawagane*) covers a core steel with low-carbon content (*Shingane*). The crystallite size distribution obtained through Bragg-edge analysis could consistently explain the internal structure of two steels observed in neutron tomograms. Our nondestructive imaging revealed deep hardening, forming a wavy pattern more than 5 mm from the cutting edge.

## Introduction

**Fig. 1:**
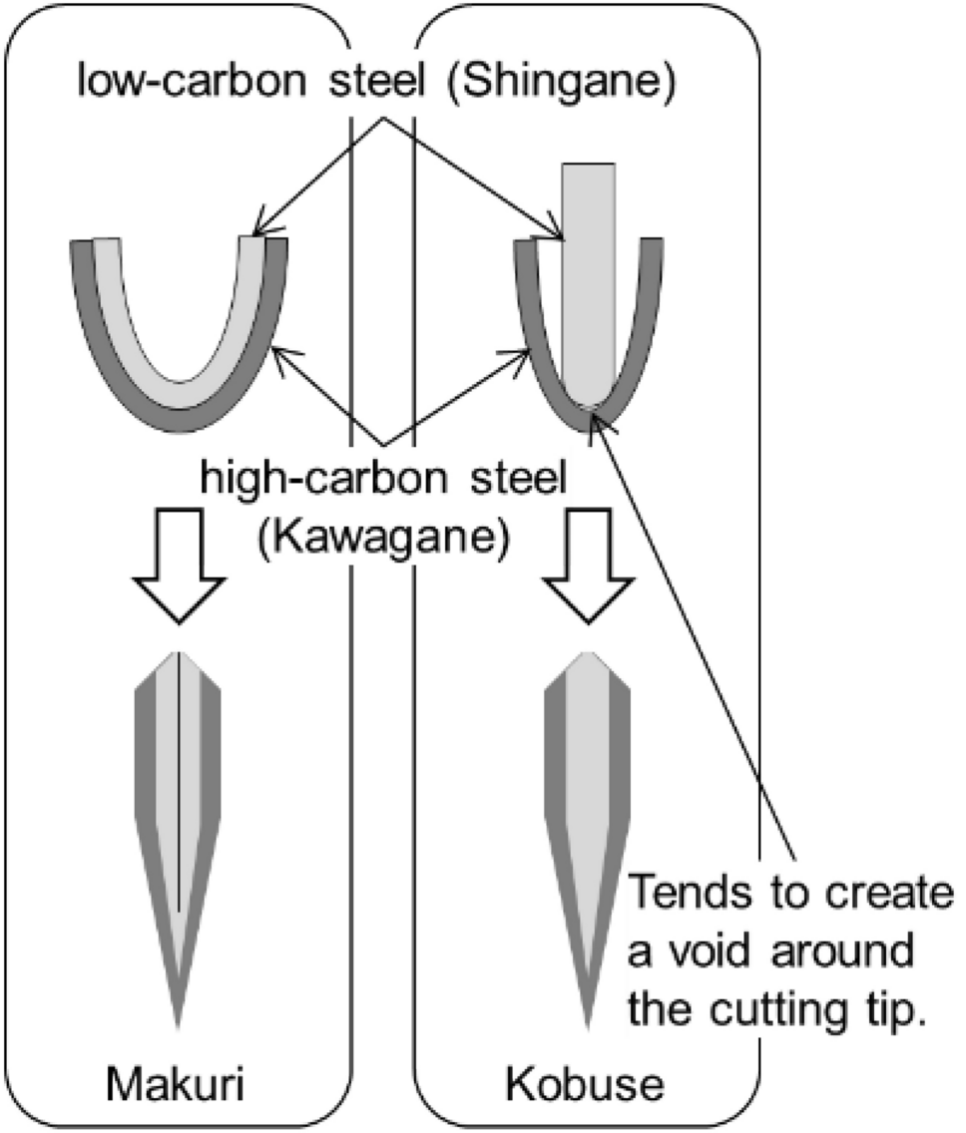
Schematic of the steel combination techniques called *Makuri* and *Kobuse* frequently seen in Bizen swords.

The distinctive appearance of the Japanese sword, now globally recognized for its artistic value, is thought to have originated around 900 AD. Japanese swords produced between then and around 1600 are referred to as *Koto* (old swords), and the details about the raw material sources and production techniques for Koto remain unknown because no surviving documents are available from that time. In the seventeenth century, Japanese swords were no longer used in actual battles until the end of Edo period and Japanese swords called *Shinto*(new swords) appeared. After the Shinto period, written documents describing their materials and manufacturing techniques survived, and the hardening of sword became deeper to make them look decorative for business purposes, even at the expense of its elasticity compared with Koto^1^. The five major sword-producing regions that flourished during the Koto period are called *Gokaden*^2^, among which Bizen (also called Bishu; present Okayama Prefecture, approximately 170 km west of Kyoto) boasted the highest production volume and number of swordsmiths and schools^3^. Norimitsu in Bizen/Bishu Osafune, whose inscription is engraved on the sword that we investigated in this study, existed from the first Norimitsu (early 1300) to approximately the end of the sixteenth century and produced several renowned Koto that exist today. Bizen swords are frequently made using a steel combination technique called *Kobuse* or *Makuri* as shown in Fig. 1^4,5^. In the Makuri method, two types of steel are forged and welded to make one plate, which is folded back and forged to make the sword shape. In the Kobuse method, an outer steel called *Kawagane* covers a core steel called *Shingane*, and they are forged and welded to make a sword shape. Forge welding around the cutting tip is particularly challenging.

The metallurgical characteristics of Japanese swords have been studied in Japan since the early twentieth century using destructive methods. Fundamental sword information was obtained, such as sword structure, metallographic structure, and carbon content^6^. During World War II, scientific investigations of Japanese swords were primarily conducted at the former Imperial Universities and Companies, focusing on their productivity and performance as weapons^1^. After the war, research shifted to public research institutions, emphasizing not only basic metallurgical research but also reproducing the beauty of Koto as works of art. Until the end of the twentieth century, these studies were primarily based on destructive testing methods. However, this period had a severe lack of documentation in English on these topics, resulting in minimal international recognition of Japanese swords.

In the twenty-first century, with the spread of the Internet, the appeal of Japanese swords became globally recognized and their historical and artistic value considerably increased. Destructive testing allows the direct observation of the internal structure of the sword and provides detailed microstructural information through various analyses. However, destroying valuable swords that have been carefully preserved for several hundred years is impossible. Recently, measurement techniques using neutrons have made substantial progress, enabling the nondestructive acquisition of information about the internal structure/properties of sword blades^2,7–14^, which must be investigated as cultural heritage items.

Norimitsu, a short sword (*Wakizashi*) was studied using Bragg-edge transmission (BET) imaging around 2015^15–17^using two counting-type two-dimensional (2D) detectors at NOBORU^18^ at the Materials and Life Science Experimental Facility (MLF) of the Japan Proton Accelerator Research Complex (J-PARC). The high-resolution images revealed coarse grains around the middle position, and the BET spectra showed preferred orientation to be somewhat strong and nonuniform. A gap (void) near the tip area was also reported. They speculated that the void was created during the outer and inner steel forge welding. However, the previous BET measurements were performed at several places on the sword, and no computed tomogram image was obtained. Therefore, overall features or changes in the characteristics of the entire sword were not revealed. Such information is valuable for considering the steel characteristics and confirming the sword structure. Furthermore, we found a void near the cutting tip of this sword; it might become a clue for the steel combination structure of the sword.

Therefore, further experiments were required. To cover the information gaps, we measured the entire blade at the energy-resolved neutron imaging system, RADEN^19^, at MLF of J-PARC. The BET imaging using a counting-type 2D detector and a new strategic neutron tomography using a camera-type detector with wavelength range longer than the Bragg cutoff energy were conducted. We also measured two additional positions using wide- and medium-wavelength neutron tomography with better resolution. We visualized a three-dimensional (3D) or 2D microstructure throughout the blade, examined the distribution of the metallurgical characteristics and elucidated that the blade was forged using the Kobuse technique of combining steels.

## Experimental

**Fig. 2:**
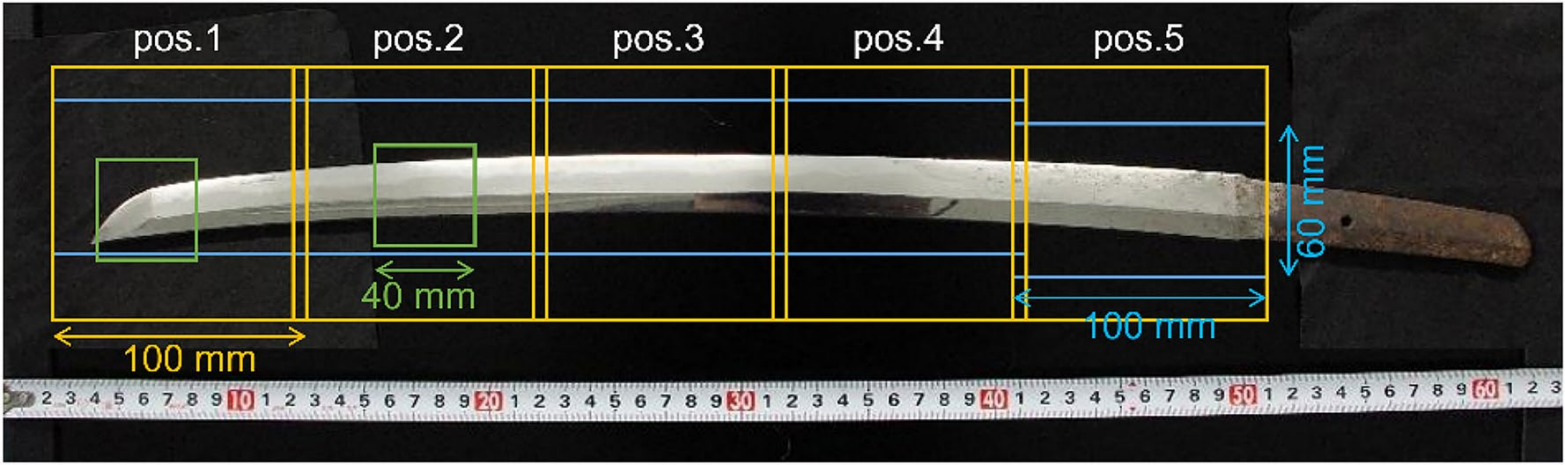
Photograph of the sword investigated in this study: blue, yellow, and green boxes indicate the field of views (FOVs) of the Bragg-edge transmission (BET) measurements, long- and wide/medium-wavelength tomography measurements, respectively.

The old sword shown in Fig. 2, Norimitsu, has a blade length of approximately 45 cm and is engraved with the signature “Bishu Osafune Norimitsu”. This sword is the personal property of Dr. K. Hirota of KEK and has been lent to Dr. Y. Kiyanagi for non-destructive analyses using various quantum beams. Norimitsu was made in the Muromachi period (1336–1573), but has been applied *Saiha*. Saiha refers to rehardening and repolishing a sword whose blade has been burned owing to a fire or other causes and has lost its original hardening pattern. Saiha swords are thought that the internal structure of the blade is not affected much because hardening is done at the end of the sword-smithing process. Neutron tomography and BET imaging were conducted at RADEN, with pulsed neutrons at 660 kW proton beam operating at 25 Hz (40 ms interval). The proton beam power was 3–4 times higher than in the previous experiments at NOBORU. Figure 2 shows a photograph of the sword and the field of view (FOV) positions for the measurements. The Japanese sword was vertically located on a rotation stage to fix/rotate the sample without remounting during all experiments. The sword was vertically stepped at 95 mm in order to slightly overlap the FOV from positions 1–5 for the BET and long-wavelength tomography. Both measurement systems were located approximately 24 m downstream from the neutron source. Herein, the diameter of the neutron beam aperture (*D*) and the distance from the aperture to the detector (*L*) (the *L*/*D* value) was ca. 400 for the tomography experiment considering spatial resolution and intensity, and ca. 720 for BET measurement considering the maximum counting rate of the detector. The neutron imaging detectors were mounted on a linear stage independent of the sample stage, and the sample-to-detector distance was approximately 50 mm for all measurements to reduce geometric image blurring.

For whole-body 3D analysis with somewhat relaxed resolution, a camera-type detector with a gated image intensifier was used for wavelength-selective neutron tomography^10,19,20^. This detector converts neutrons into visible light using a 0.1-mm thick ^6^LiF/ZnS scintillator screen (RC Tritec AG) and generates 16-bit 2048 × 2048 pixels Tag Image File Format (TIFF) images using a cooled charge-coupled device (CCD) camera (Andor Technology Ltd.). For the measurement, the timing of optical amplification by the intensifier was limited to a time-of-flight (TOF) from 26 to 39 ms using the gating function, a neutron wavelength range of 4.3–6.5 Å (indicated by a double-headed arrow in Fig. 4(b)) was selected, and the FOV was ca. 100 × 100 mm^2^ with ca. 50 μm pixel size. In this measurement, three projection images were acquired at one angular condition with a camera exposure time of approximately 25 s (550 proton pulses) and then averaged to improve statistical accuracy and remove the background signals from the readout noise of the CCD camera. Furthermore, the distribution of incident neutron intensity was normalized using the image without the sample. The 2D spatial resolution under these experimental conditions was approximately 170 μm at the sample rotation center. The sample was rotated 360° in 1° increments to meet the 2D spatial resolution and empirically expected resolution after 3D reconstruction. The cross-sectional images (tomograms) were reconstructed from the 360 projection images using the filtered-back-projection algorithm in the visualization software VGSTUDIO MAX (Volume Graphics GmbH).

Additional neutron tomography was conducted at RADEN with better resolution using pulsed neutrons at 710 kW proton beam. This time, a wide-wavelength range was used to gain neutron flux. The same CCD camera using a 0.05-mm thick ^6^LiF/ZnS scintillator screen (RC Tritec AG) was used; however, the image intensifier was not used because it somewhat degrades the spatial resolution. The disk-chopper system was used for the measurement and tuned to select either a wide neutron wavelength range of less than 8.6 Å or a medium neutron wavelength range of 1.5–4.0 Å (as indicated by a double-headed arrow in Fig. 10(b)), and the FOV was ca. 40 × 40 mm^2^ with ca. 20 μm pixel size. The sample was rotated 360° in 0.6° increments to obtain the entire dataset. Each projection image was acquired at a camera exposure time of approximately 255 s (6000 proton pulses). As the statistical accuracy under this measurement condition is sufficiently high, only one projection image was acquired at each angular position. Background processing and normalization with an open beam were performed using the same method described above. The 2D spatial resolution under these experimental conditions was approximately 70 μm at the sample rotation center. Table 1 summarizes the neutron tomography measurement conditions.

**Table 1:** Summary of the neutron tomography measurement parameters.

Wavelength range	Gate device	Scintillator type	Macro lens	FOV (mm^2^)
4.3–6.5 Å (long-wavelength)	Image intensifier	^6^LiF/ZnS 100 μm	50 mm *f*/1.2	100 × 100
< 8.6 Å (wide-wavelength)	Disk chopper	^6^LiF/ZnS 50 μm	150 mm *f*/2.8	40 × 40
1.5–4.0 Å (medium-wavelength)	Disk chopper	^6^LiF/ZnS 50 μm	150 mm *f*/2.8	40 × 40

A high-count rate of 1 mega-counts-per-second (Mcps) 2D detector, a boron-type micropixel chamber-based neutron imaging detector (B-µNID)^21^, was used for BET imaging. The detector had a spatial resolution of ca. 0.35 mm and a maximum measurement area of 102.4 × 102.4 mm^2^. The TOF was converted to the wavelength using the flight path length from the neutron source to the detector, which was determined to be 23.878 m using the standard iron sample data. During the BET measurement, the neutron wavelength range was 1.0–6.5 Å (Fig. 4b) using the disk-chopper system, and the FOV was approximately 60 mm in width and 100 mm in height using a B_4_C slit at upstream of the sample. The geometric image blurring of ca. 0.07 mm was sufficiently smaller than the spatial resolution of the B-µNID. Event data were accumulated for approximately 7 h for each position, and transmission images in TIFF were generated from the event data using a 0.4 mm pixel size and 10 µs time bins.

Furthermore, after the sample was moved out of the FOV, an open-beam image reflecting the intensity distribution of incident neutrons was similarly measured. This open-beam image was used for normalization to derive position-dependent BET spectra. Sufficient data statistics for the analysis were obtained by summing the BET spectra for the 3 × 3-pixel (1.2 × 1.2 mm^2^) regions into a single BET spectrum, and the pixel area was stepped at 1-pixel (0.4 mm) intervals in the *x*- and *y*-directions. All BET spectra were analyzed using a Rietveld-type profile-fitting analysis code, the Rietveld Imaging of Transmission Spectra (RITS)^22–24^. In all analyses and discussions in this paper, the crystal structure of steel (ferrite/martensite phase) is assumed to be a single-phase body-centered-cubic (BCC) structure. The analysis does not include cementite in pearlite and retained austenite because their contribution to the BET spectra is negligible.

**Fig. 3:**
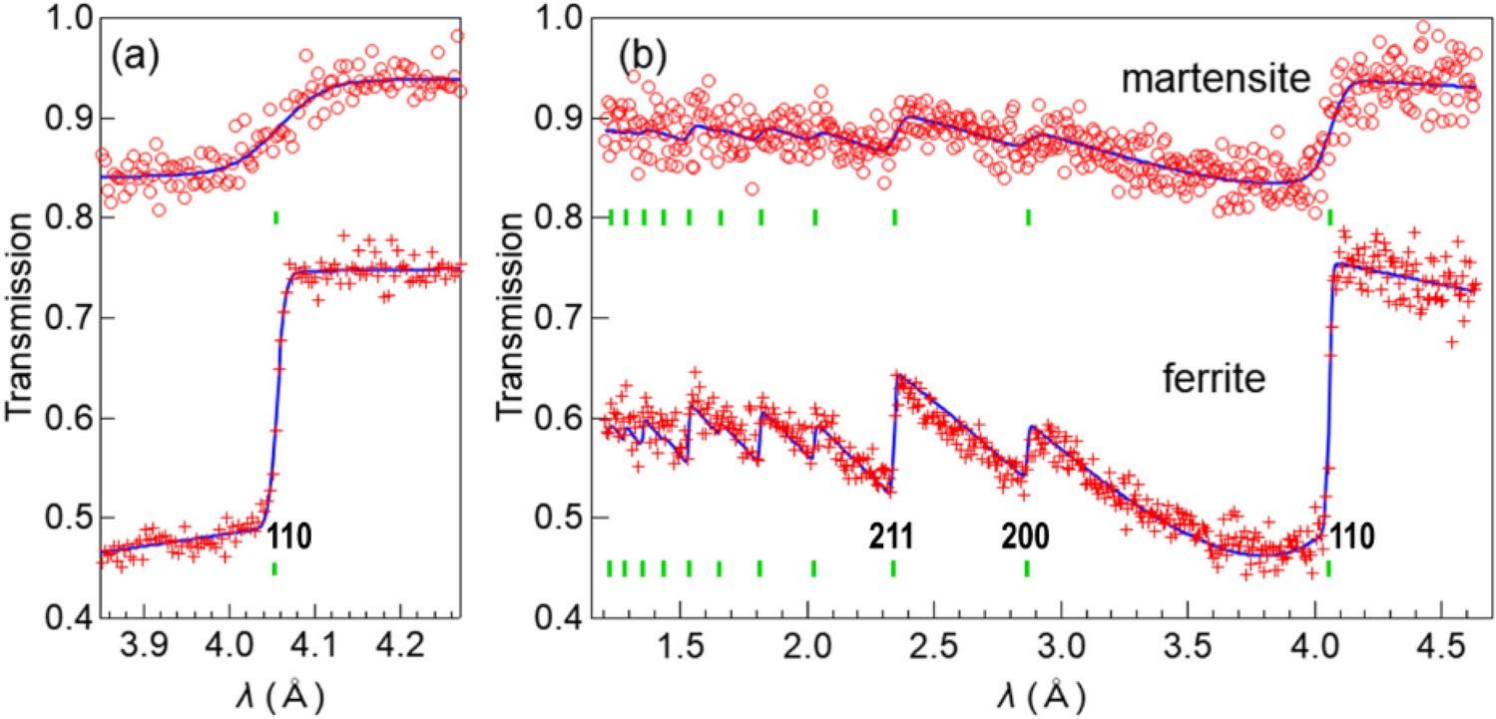
Examples of (**a**) single-edge and (**b**) full-pattern analyses of the ferrite and martensite phases of Norimitsu.

A single-edge analysis^25^ was performed to obtain the (110) lattice plane spacing *d*_110_ and Gaussian profile parameter for the (110) edge *σ*_110_ using the wavelength range of 3.81–4.31 Å. Examples of single-edge analysis for the ferrite and martensite phases in Norimitsu are shown in the Fig. 3(a). The hardening region of the cutting edge shows a high transmission due to its thin thickness, and a broad edge pattern due to the martensite phase formation. The rest of the hardened area has thickness of several millimeters and consists of ferrite/pearlite phase, resulting in a sharp edge pattern. The edge broadening *w*_110_ was obtained from *σ*_110_ by subtracting the instrumental resolution contribution using standard iron sample data. A full-pattern analysis was performed using the wavelength range of 1.20–4.65 Å to obtain the projected atomic number density *ρt*, crystallite size *s*, and preferred orientation parameter *r*. The primary extinction effect correction employed in the RITS code is the Sabine function^26–28^, which is widely used in Rietveld analysis for neutron powder diffraction (NPD) data, whose parameter *s* provides the crystallite size in μm. For the present data analysis, the crystallite size parameter was either fixed to 0 or refined from 1 or 3 as the initial value, and the one minimizing the residual sum of squares was selected by comparing the results. The preferred orientation correction used in the RITS code is an extension of the March–Dollase function widely used in Rietveld analysis for NPD data. In the NPD data or BET spectrum, the crystal orientation distribution information on the Debye–Scherrer ring is integrated and averaged, resulting in loss of the information. Therefore, an axially symmetric crystal orientation distribution is assumed with respect to the incident beam direction. Under such conditions, the pole figure and the March-Dollase function are equivalent^28,29^. In the RITS code, the preferred orientation vector is assumed as the most strongly oriented crystal plane, and the degree of crystal orientation anisotropy (degree of texture development) is obtained as a parameter *r* of the March–Dollase function. Examples of full-pattern analysis for the ferrite and martensite phases in Norimitsu are shown in Fig. 3(b). For the present data analysis, assuming a < 320 > preferred orientation vector yielded good overall fitting results.

## Results and discussion

### Energy-selective image obtained using the B-µNID

**Fig. 4:**
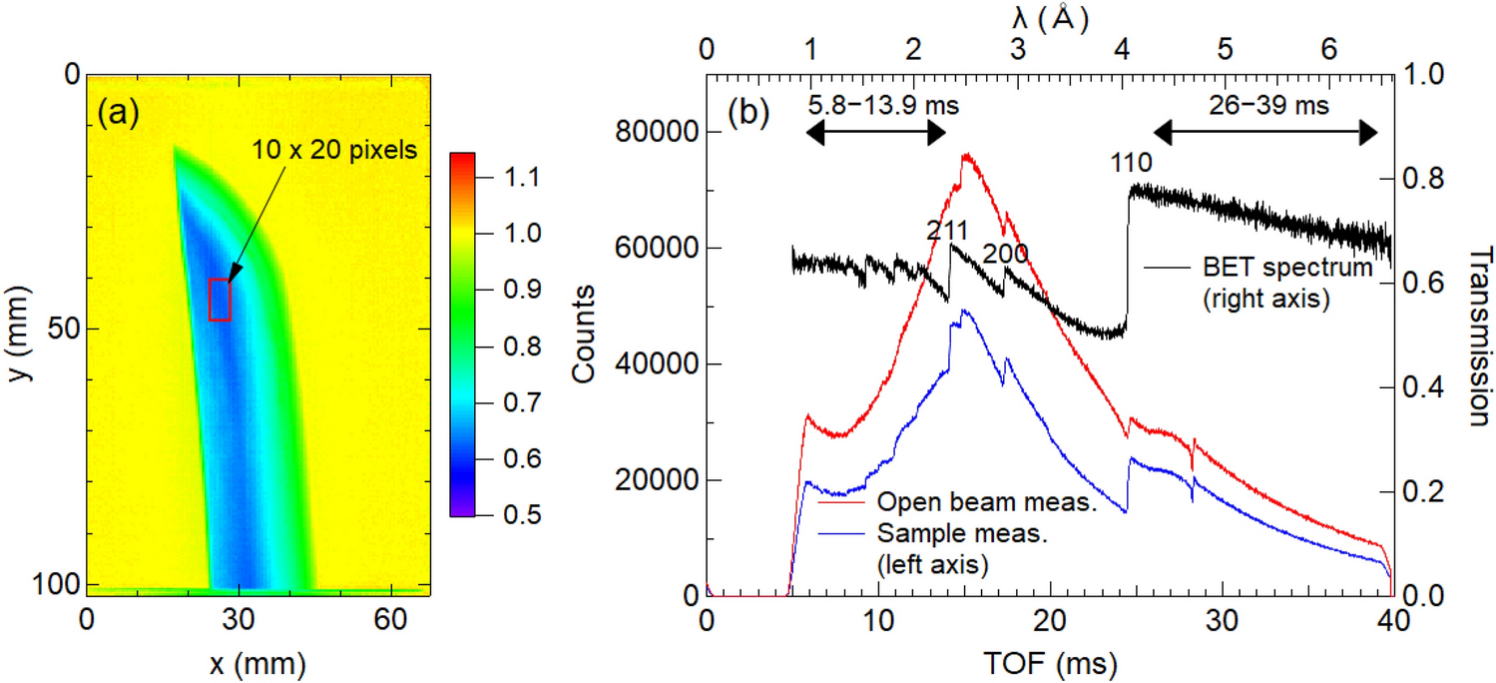
(**a**) Normalized image of the transmission data measured with the boron-type micropixel chamber-based neutron imaging detector (B-µNID) at position 1; (**b**) time-of-flight (TOF)/lambda spectra of the sample (blue) and open beam (red) obtained by summing the data of 10 × 20 pixels shown in (**a**) and corresponding BET spectrum (black).

The transmission image was obtained by normalizing the transmission of outside the sample image to 1.0 using the open-beam image. Figure 4(a) shows an example of a transmission image at position 1 using the wavelength range of 1.0–6.5 Å. The color scale was chosen to make it easy to see the color change around 1.0. Figure 4(b) shows the TOF/lambda spectra obtained by summing the data of 10 × 20 pixels, surrounded by the red square near the center of Fig. 4(a). The red and blue lines are the scaled open beam and the sample transmission spectra, respectively, and the black line is the BET spectrum obtained by their ratio. The red and blue lines include Bragg edges of aluminum windows present in the incident beam path, but these edges are canceled correctly in the BET spectrum. The Miller indexes for the (110), (200), and (211) edges are shown in the figure.

**Fig. 5:**
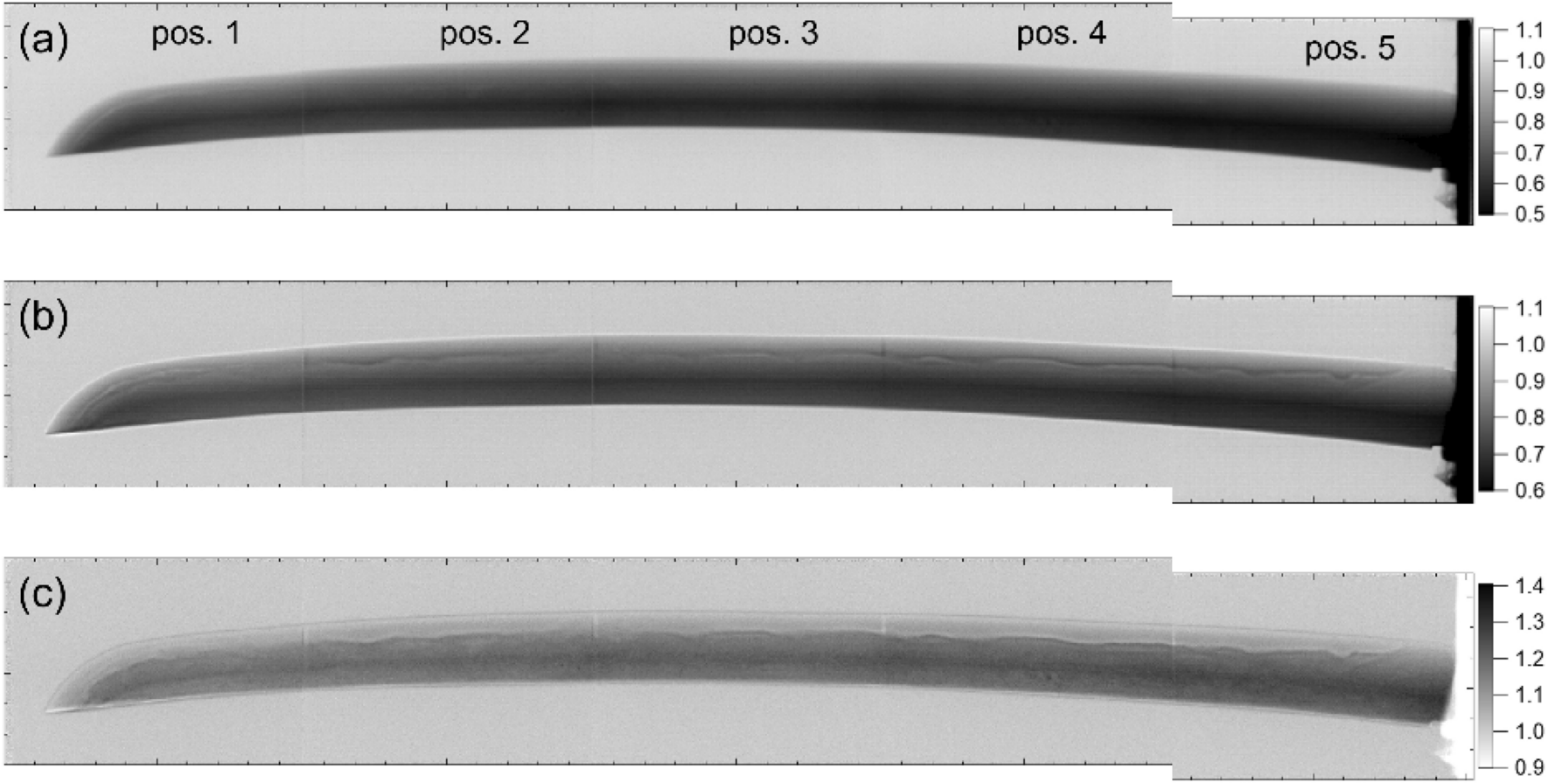
Normalized gray-scale images of (**a**) 5.8–13.9 ms (short wavelength: 0.96–2.3 Å) and (**b**) 26–39 ms (long wavelength: 4.3–6.5 Å) and (**c**) their ratio ((**b**)/(**a**)) extracted from the data measured with the B-µNID; the ticks on the axis are marked at 5 mm intervals.

Figure 5 shows the normalized gray-scale image of (a) 5.8–13.9 ms (short wavelength: 0.96–2.3 Å) and (b) 26–39 ms (long wavelength: 4.3–6.5 Å) extracted from the data measured using the B-µNID. The double-headed arrows in Fig. 4(b) indicate these ranges. In addition, the image ratio of (b)/(a) is shown in (c). In Fig. 5(a), the contrast reflects the sample thickness, whereas in Fig. 5(b), a region with a transmission exceeding 1.0 appears on the outer periphery of the blade and the additional contrast modulation can be seen near the hardening boundary. If these contrasts originate from absorption or Bragg scattering, it should appear in Fig. 5(a); however, it is not visible, no matter how the scale is adjusted. These contrasts are enhanced in Fig. 5(c) in the form of a more pronounced temper pattern on the cutting-edge side. A slight increase in transmission originates from voids in the tip area can be seen by adjusting the scale in Figs. 5(a) and 5(b). Furthermore, contrasts caused by changes in the degree of preferred orientation or coarse grains appear if an appropriate wavelength is selected in the Bragg-scattering region.

As expressed by the formula of the BET spectrum^22^, Bragg scattering does not occur at wavelengths longer than the (110) edge of the ferrite/martensite phase (the Bragg cutoff phenomenon). Therefore, the contrast only appearing in Fig. 5(b) is due to the difference in larger-scale interaction than Bragg scattering, which is enhanced in the cold neutron region. Although their physical origin is currently unknown, some interaction phenomenon shown in Fig. 5(b) is used in our wavelength-selective neutron tomography to amplify the contrast in the hardening region and the interior of the blade.

### Energy-selective neutron tomography

**Fig. 6:**
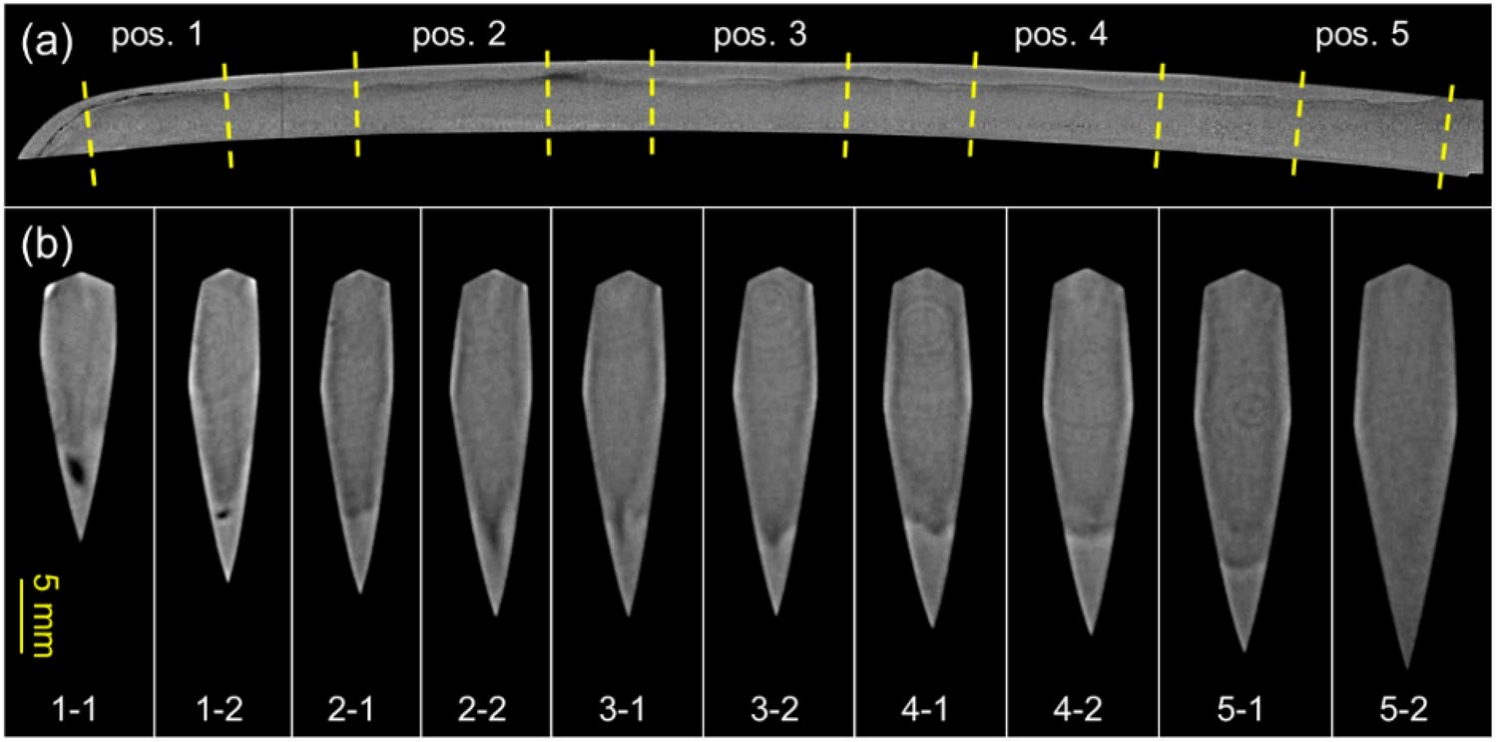
(**a**) Longitudinal tomogram (parallel to the blade) and (**b**) transverse tomograms (perpendicular to the blade) by the long-wavelength neutron tomography. The contrast observed in the quenched area is not due to the density difference of the steel.

Figure 6 shows the tomography analysis results of Norimitsu. The longitudinal tomogram in Fig. 6(a) represents the entire cross-sectional image parallel to the blade. This image was obtained by averaging tomograms of the center of the blade with 0.2-mm thickness. The hardened area is visible in this longitudinal tomogram, and the boundary shape is similar to the contrast observed in Fig. 5(b). In contrast, by averaging tomograms with a length of approximately 3 mm, we obtained transverse tomograms at 10 locations, indicated by the yellow-dotted lines in Fig. 6(a). Figure 6(b) shows these cross-sectional images perpendicular to the blade. The dark-colored areas inside the blade indicate low scattering density. As discussed later, the black areas on the cutting tip of 1–1 and 1–2 are voids caused by failed forge welding. The bright areas in the cutting edge indicate the martensite phase formed by the hardening. In addition, from 1–2 to 4–2, the external shape of the sword blade is asymmetrical. The contrast in the cross-sectional view, such as 3–1, indicates that the left-hand side of the sword might have been heavily resharpened from the original blade shape. The transverse tomogram 5–2 shows the part that has not been hardened, and almost no contrast is observed except for the widest part, which is slightly bright owing to artifacts. The slightly bright, thin layer observed near the maximum width of the cross-sectional view from 3–2 to 4–2 differs from the artifact in 5–2, indicating that it captures the layered steel structure with different carbon content; however, this is yet to be confirmed.

**Fig. 7:**
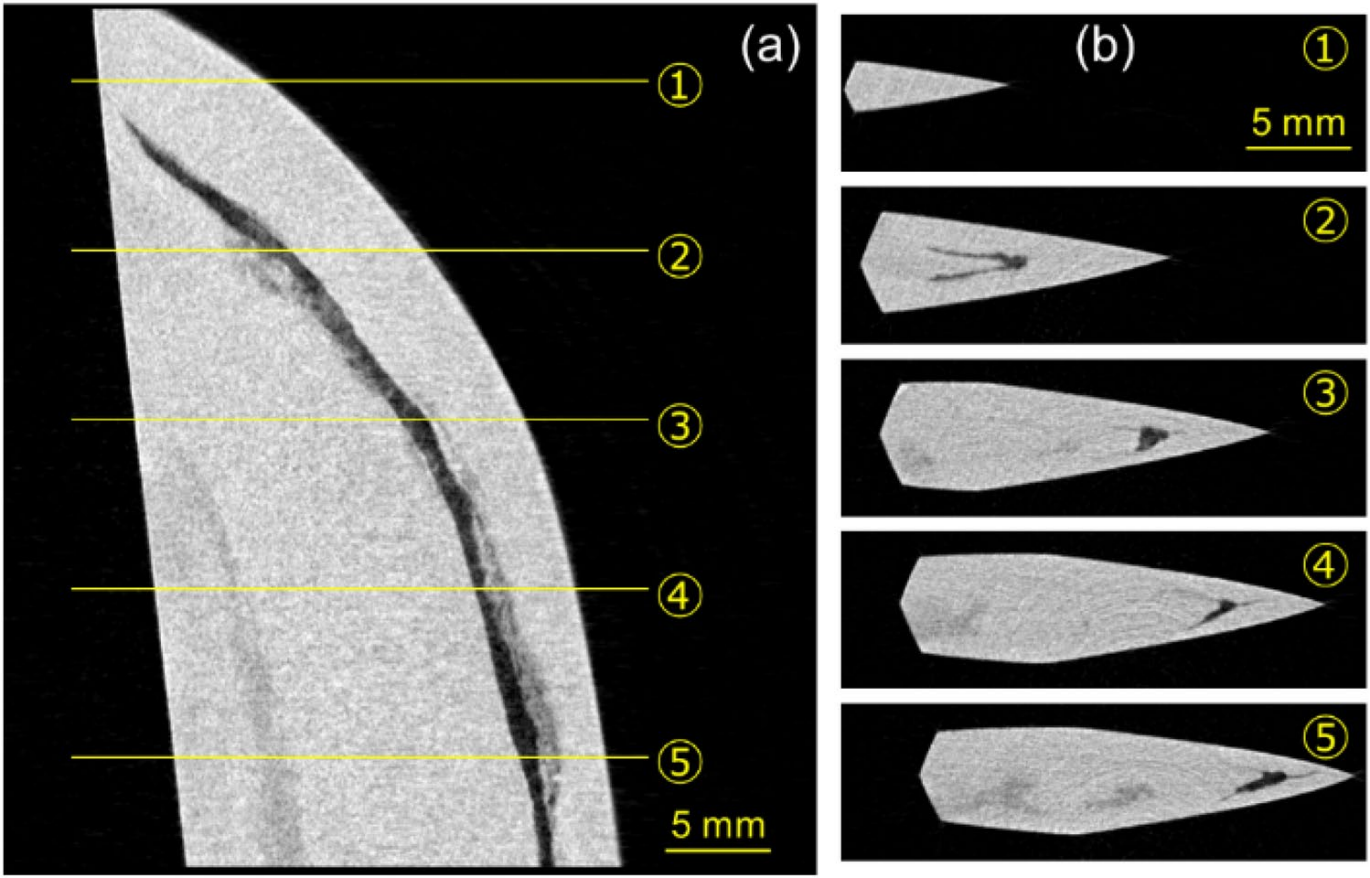
Cross-sectional tomograms of the cutting tip (**a**) parallel (longitudinal tomogram) and (**b**) perpendicular (transverse tomogram) to the blade by the wide-wavelength neutron tomography. The quenched area does not exhibit contrast because of the small density difference of the steel.

A careful examination of the 3D tomogram of position 1 reveals a continuous and gradual change in the shape and position of the void over a span of approximately 10 cm. Therefore, we performed a better spatial-resolution tomography experiment with a resolution of 70 μm. In this additional measurement, a wide-wavelength range was used to gain neutron flux to compensate for the reduced effective f-number of the macro lens or the sensitivity of the imaging system. Furthermore, all tomograms are output with 0.05-mm thickness. A distinctive defect was observed over a wide area at the cutting tip. Figure 7(a) shows a longitudinal tomogram of the center of the blade with 0.05-mm thickness. Figure 7(b) shows transverse tomograms on the cutting tip, showing cross-sectional images along a length direction stepped at 8 mm with 0.9-mm thickness. One should note that the density difference between the ferrite/pearlite phase and the martensite phase in steel is approximately 0.4%. As shown in the cross-sectional tomograms in Fig. 7, the quenched area does not exhibit contrast due to the density difference of the steel. On the other hand, the contrast observed in the quenched area in Fig. 6 is not due to the density difference of the steel. In Fig. 7, the void structure shown in Fig. 6 is captured clearly at higher resolution. In particular, the tomograms in Fig. 7(b) capture the V-shaped boundary region between the core and outer steel. Furthermore, as seen from the transverse tomograms of ④ and ⑤, the outer steel, except for the cutting edge, is much thinner. Thus, the slightly bright, thin layer seen in Fig. 6(b) is presumed to be the outer steel. Japanese swords made with the Kobuse and Makuri techniques (Fig. 1), featuring a layered structure with low-carbon steel at the core and high-carbon steel on the outside, are difficult to distinguish. However, the state of void formation in the internal structure of the blade observed by tomography analysis indicates that Norimitsu was made using the Kobuse technique because this defect structure characteristically occurs during forge welding using the Kobuse technique rather than the Makuri technique.

### Bragg-edge transmission imaging

**Fig. 8:**
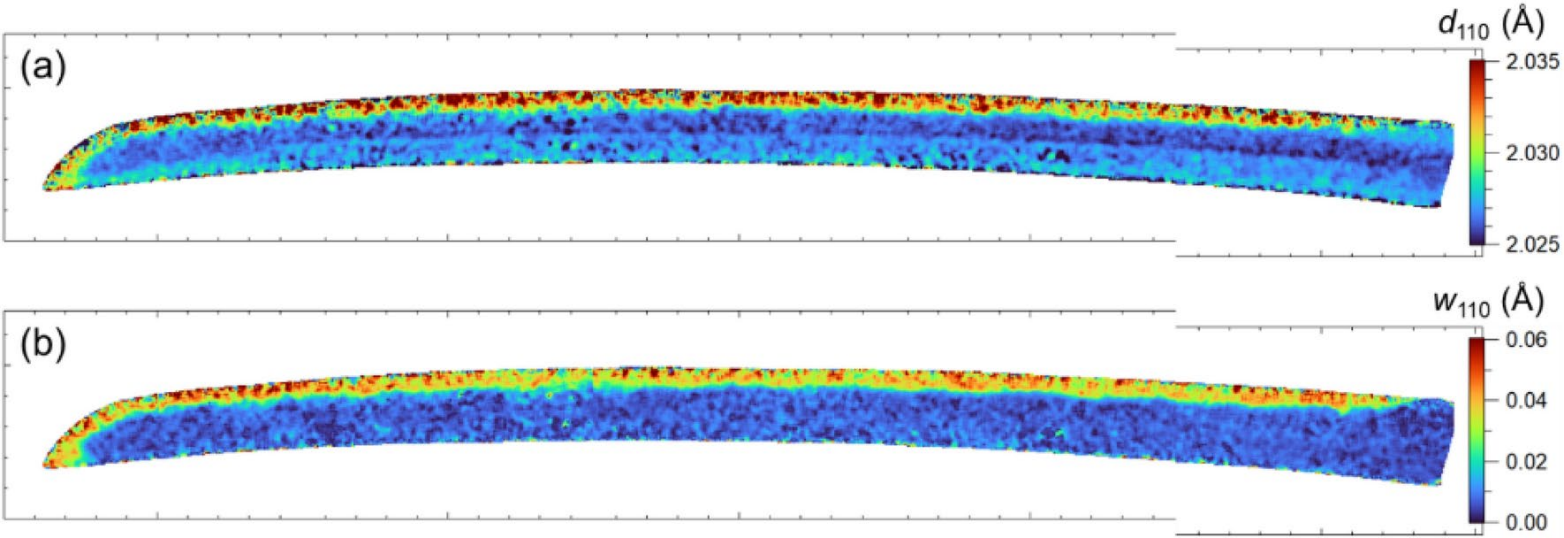
Mapping of (**a**) the (110) lattice plane spacing *d*_110_ and (**b**) edge broadening *w*_110_; the ticks on the axis are marked at 5 mm intervals. The hardening region is clearly visible in both maps.

**Fig. 9:**
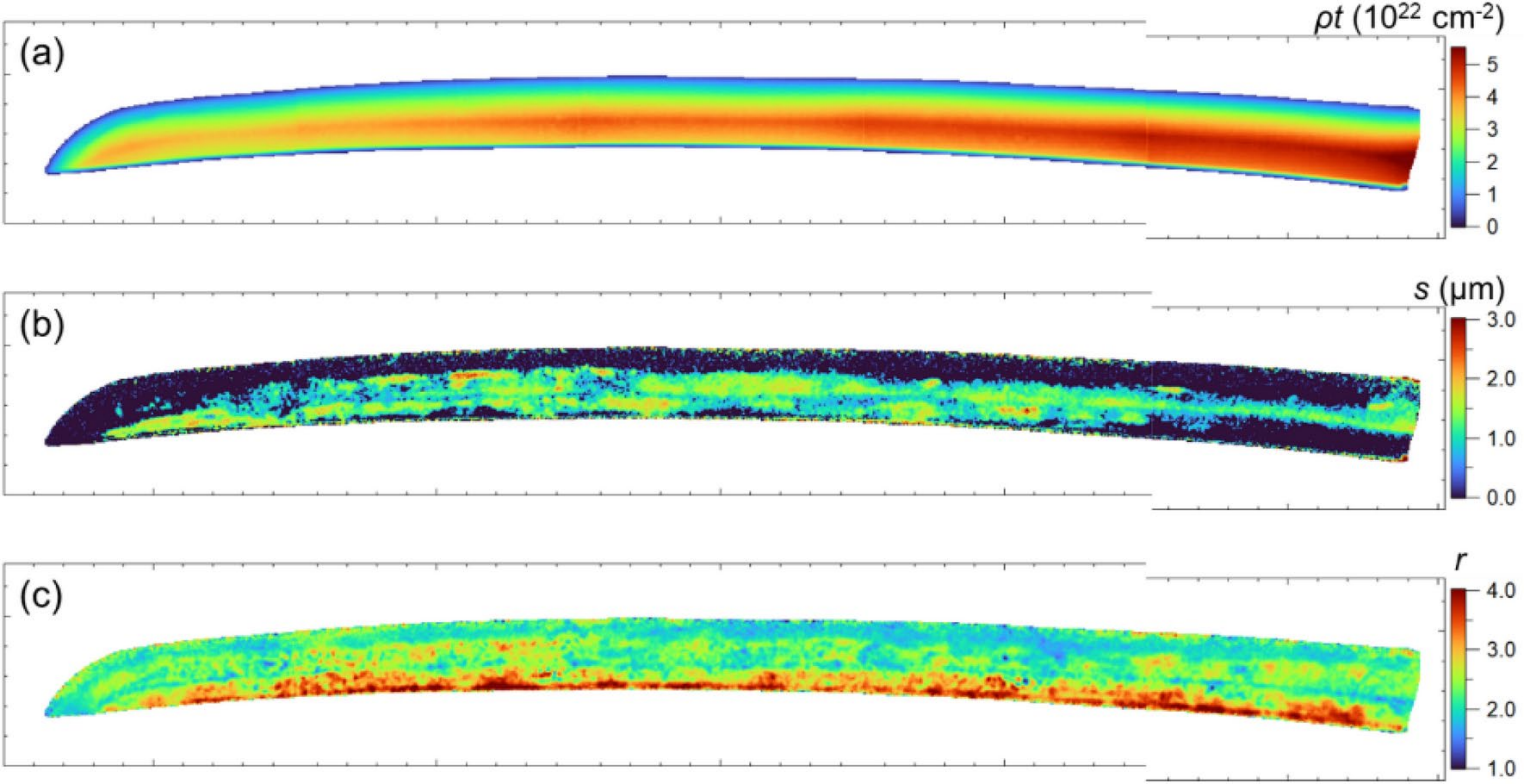
Mapping of (**a**) the projected atomic number density, (**b**) crystallite size, and (**c**) degree of preferred orientation; the ticks on the axis are marked at 5 mm intervals. On the back side, the crystallite size is large and the degree of preferred orientation is considerably strong.

Figures 8 and 9 summarize the BET analysis results of Norimitsu. As shown in Fig. 2, the BET spectra were taken for the entire blade by moving the FOV from the cutting tip at position 1 to the blade end at position 5, just as in the tomography measurement.

Lattice spacing *d*_110_ and edge broadening *w*_110_ of the (110) plane shown in Figs. 8(a) and 8(b) increase at the cutting edge, indicating the formation of the hardened phase, such as the lath martensite owing to quenching^30^. The martensite phase is widely distributed in a wavy pattern along the cutting edge. This trend is more clearly shown in the neutron tomograms. Old swords we have investigated so far are not deep hardened^10,12,13^; however, our present analysis has revealed that Norimitsu is deep hardened. This feature could be explained that Norimitsu’s Saiha was probably conducted in the trend of the Shinto period and later.

Figure 9(a) shows the mapping of the projected atomic number density (*ρt*) of iron, assuming the BCC structure. The obtained value is proportional to the sword thickness. The void at the cutting tip can be found by adjusting the scale for this purpose, however, it is hard to see with the scale in Fig. 9(a). Figure 9(b) shows the mapping of the crystallite size distribution of Norimitsu. The crystallite size obtained by the Sabine function is evaluated by the primary extinction effect (re-diffraction phenomenon) within a perfect crystal block (the mosaic block). The evaluated crystallite size of steel is smaller than, but proportional to, the grain size estimated from optical microscopy^22^. Recent studies have shown that there is a proportional relationship between the crystallite size and grain size estimated from electron backscatter diffraction, as well^31^. In Japanese swords, the crystal grain size of low-carbon steel (ferrite) at the core is larger than that of high-carbon steel (pearlite and ferrite) on the outside^30^, indicating that the crystallite size has a similar tendency, although the absolute value is much smaller due to some kind of defects and may coincide with the sub-grain size^27^. This figure reveals that fine crystallites of approximately 0.5 μm or less are distributed on the cutting-edge side where the blade is thin. In other areas, crystallite sizes of 1–2 μm are widely observed from the back ridge of the blade to approximately 2/3 of the blade width, except for the blade end area. This result can be approximately explained by considering that the BET reflects the volume-averaged values of the low- and high-carbon steel along the transmission direction. The observed crystallite size distribution of Norimitsu, large on the back side of the sword, is almost consistent with the same region and similar to the trend observed in previous measurements of the Bizen’s old sword^13^. Figure 9(c) shows the mapping of a parameter *r* of the March–Dollase function assuming a preferred orientation vector < 320 > , which is relatively close to the vector < 110 > in the inverse pole figure. The *r* values are greater than 1 across most regions, indicating that the assumed preferred orientation vector for ferrite/martensite is perpendicular to the incident beam direction. In the back ridge region, where the value exceeds 2.5, the evolution of the preferred orientation (texture) is considered strong.

**Fig. 10:**
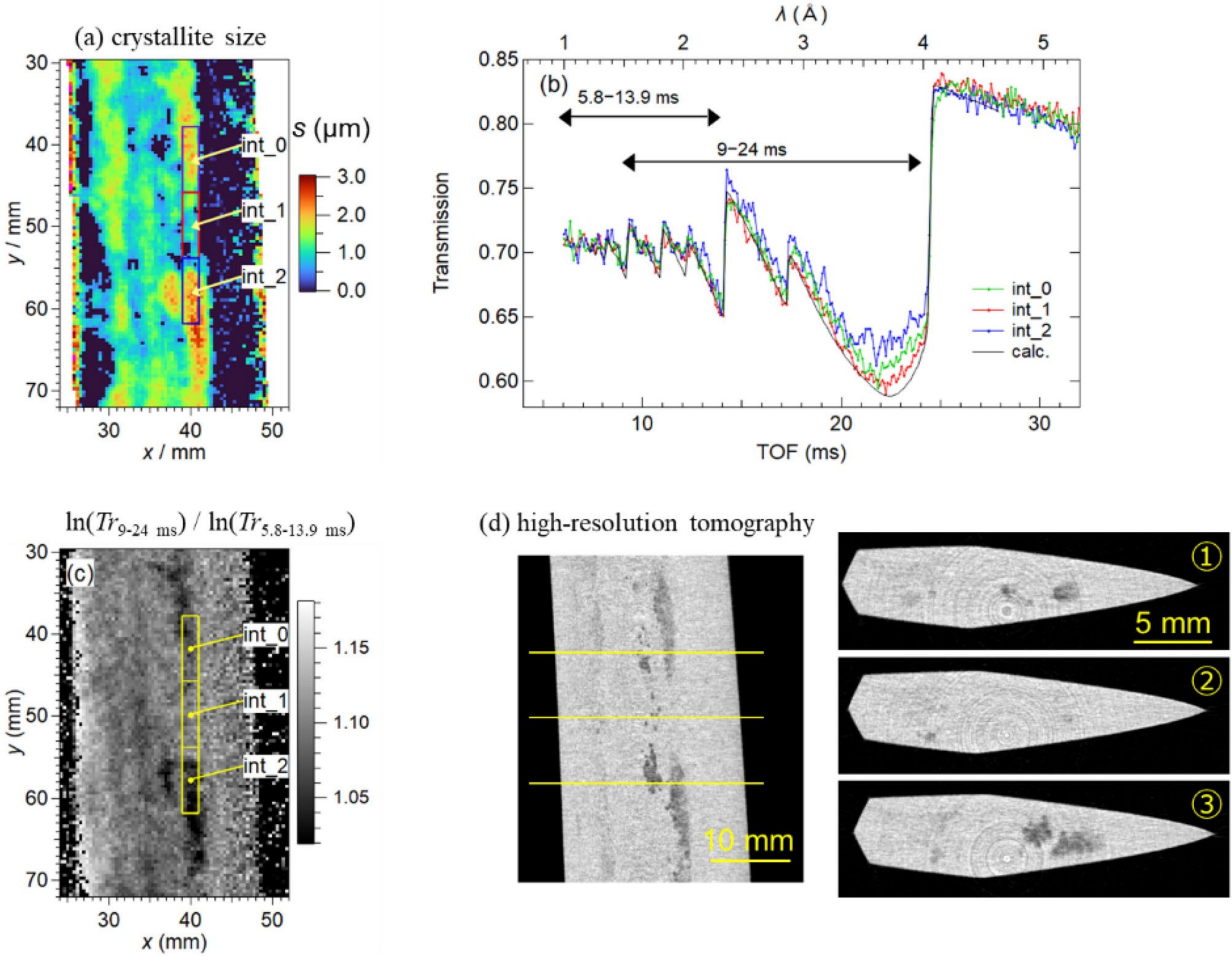
(**a**) Enlarged map of the crystallite size at position 2, (**b**) normalized BET spectra of the selected areas indicated by the three rectangles in (**a**) and a calculated BET spectrum, (**c**) the wavelength-range contrast imaging of the same region as (**a**), and (**d**) the cross-sectional images by high-resolution medium-wavelength neutron tomography.

In a previous paper^16^, the Bragg edge imaging experiment using a high-spatial-resolution detector with a 55 μm pixel^32^ revealed coarse grains around position 2, and dip-like transmission spectra were observed. We reinvestigated this position with high-resolution 3D tomographic analysis and further investigated the 3D distribution of coarse grains in detail by combining it with 2D BET data analysis of the same location. The BET spectra containing coarse grains deviate from the powder/bulk conditions assumed in the RITS function; therefore, the absolute crystallite size values cannot be correctly obtained; however, trends in the relationship between large and small crystallite sizes can be obtained. Figure 10(a) shows the enlarged mapping of the crystallite size at measurement position 2. The BET spectra of the selected areas indicated by the three rectangles in Fig. 10(a) are shown in Fig. 10(b). The BET spectra of int_0 and int_2 are normalized to int_1 using their respective *ρt*, and the calculated BET spectrum is plotted as “calc.” based on the parameters obtained from RITS fitting for int_1 with its crystallite size parameter to be zero. The spectra of int_0 and int_2 exhibit a large primary extinction effect at 2.5–4 Å, resulting in a large value in the crystallite size map (Fig. 10(a)).

The wavelength (λ)-dependent neutron transmission spectrum of a crystalline sample is expressed as follows^22^:

1 $$Tr\left(\lambda \right)={\text{exp}}\left(-\Sigma_{p}{\sigma }_{\text{tot,}p}\left(\lambda \right){\rho }_{p}{t}_{p}\right),$$

where *σ*_tot,*p*_(*λ* or *TOF*) is the wavelength-dependent neutron total cross-section, *ρ*_*p*_ is the projected atomic number density, and *t*_*p*_ is the thickness of the crystalline phase *p*. Here, we tentatively define *Tr*_9–24 ms_ and *Tr*_5.8–13.9 ms_, where the former and latter are the wavelength-selective transmissions averaged over 9–24 and 5.8–13.9 ms, respectively. A wavelength-range contrast image is obtained using the ratio ln(*Tr*_9–24 ms_)/ln(*Tr*_5.8–13.9 ms_), where the dependence on *ρ*_*p*_*t*_*p*_ is canceled. Therefore, Fig. 10(c) represents the ratio of the wavelength-range-dependent *σ*_tot,*p*_, i.e., *σ*_9–24 ms_/*σ*_5.8–13.9 ms_. The result in Fig. 10(c) indicates that the coarse grains decrease *σ*_tot,﻿*p*_ and increase the transmission. High-spatial-resolution neutron tomography using a similar neutron wavelength range was conducted to capture the distribution of these coarse grains in 3D. The medium-wavelength range of 1.5–4.0 Å (double-headed arrows of 9–24 ms in Fig. 10(b)) was chosen using the disk chopper of RADEN. Figure 10(d) shows the tomography analysis results, where the dark-colored areas inside the blade indicate lower scattering density. Figure 10(c), obtained by BET imaging, and the longitudinal tomogram of Fig. 10(d), obtained by high-resolution tomography, exhibit similar contrast distributions. Therefore, the darker areas in the transverse tomograms capture areas of the coarse grains where transmission increases owing to the primary extinction effect. The internal distribution of coarse grains revealed in this way may indicate that the forging process was somewhat rough overall.

## Conclusions

This study nondestructively evaluated the structural features associated with the sword-making process of Norimitsu using long-wavelength/high-resolution neutron tomography and BET imaging. The 2D distribution of the microstructure, the 3D hardened structure at the cutting edge, and enveloping layered structure of the sword blade have been visualized.

The void shape, which remains approximately 10 cm around the cutting tip, strongly indicates that this defect resulted from the Kobuse technique, commonly used in Bizen swords. The crystallite size distribution obtained by the BET method and the contrast seen throughout the blade in the computed tomogram image is also consistent with a layered structure of Kobuse, which is composed of low carbon steel on the inside and high carbon steel on the outside. As structural characteristics are considered to be unaffected by the Saiha process, these features remain as they were when the sword was made in Koto period.

## Data Availability

The datasets used and/or analysed during the current study available from the corresponding author on reasonable request.
